# The natural human adaptive IgG-specific immune response is skewed towards non-protective tail domains of DNABII proteins

**DOI:** 10.3389/fimmu.2026.1694547

**Published:** 2026-02-27

**Authors:** Kathryn Q. Wilbanks, Jaime D. Rhodes, Steven D. Goodman, Sanjay Sethi, Timothy F. Murphy, Lauren O. Bakaletz

**Affiliations:** 1 Center for Microbe and Immunity Research, Abigail Wexner Research Institute at Nationwide Children’s Hospital, Columbus, OH, United States; 2 Department of Pediatrics, The Ohio State University College of Medicine, Columbus, OH, United States; 3 Department of Veteran Affairs Western New York Health System, Buffalo, NY, United States; 4 Division of Pulmonary and Critical Care Medicine of the Department of Medicine, University at Buffalo, State University of New York, Buffalo, NY, United States; 5 Division of Infectious Diseases, University at Buffalo, State University of New York, Buffalo, NY, United States

**Keywords:** active immunization, bacterial pathogenesis, biofilm, HU, IHF, immune redirection, original antigenic sin

## Abstract

Biofilm-mediated infections are highly recalcitrant to antibiotics and host immune system clearance. The matrix that envelopes biofilm-resident bacteria is stabilized by extracellular DNA as well as integrated ubiquitous bacterial DNABII proteins that exhibit potential as a common therapeutic target. We’ve shown that a monoclonal antibody directed against the immunoprotective DNA-binding ‘tips’ of DNABII proteins disrupts diverse biofilms, prevents their formation, and augments disease resolution in four distinct pre-clinical models. As an immunogen, a synthetic peptide that mimics the immunoprotective domains induces a response with equivalent effectiveness. Confoundingly, however, sera from both children with chronic otitis media (OM) and healthy adults preferentially recognize non-protective ‘tail’ domains of DNABII proteins. Thus, we wondered if this is a universal, natural immune response that contributes to biofilm recalcitrance. Here, we used both surface plasmon resonance and ELISA assays to assess sera from 16 healthy children, 16 additional children with chronic OM, 15 adults with chronic obstructive pulmonary disease, and 30 people with cystic fibrosis for relative recognition of synthetic peptides that mimicked either the protective or the non-protective DNABII protein domains. In 74 of 77 sera assessed (96%), we found significant IgG-specific preferential immune recognition of non-protective domains. These new data support continued development of our DNABII-directed vaccine candidate designed to re-direct the immune response toward the protective domains to mediate biofilm eradication.

## Introduction

Diseases in which biofilms are implicated to play a significant role in pathogenesis and account for approximately 80% of all chronic and recurrent bacterial diseases (1). The biofilm lifestyle is responsible for the marked recalcitrance of many diseases to treatment by conventional antibiotics and/or eradication by the host’s immune system (2, 3). This recalcitrance is due, in part, to multiple characteristics of the self-produced extracellular polymeric substance (EPS) that envelops and imparts architectural structure, shelter, and mechanical stability to biofilm-resident bacteria (4, 5). The biofilm EPS is comprised of many constituents including nucleic acids and extracellular DNA (eDNA), lipids, polysaccharides, and various proteins, the exact composition of which varies depending on the resident microbial community, host environment, local shear stress, and availability of nutrients (6). Thus, development of approaches designed to target and eradicate specific components of the EPS can be complicated and challenging (7, 8). Identification of a common EPS constituent that could potentially serve as a powerful target for more universally effective therapeutic or preventative strategies would be of great benefit, particularly given that biofilm-mediated diseases present a notable global socioeconomic burden that is estimated to be upwards of $280 billion (9).

An additional and abundant component of the biofilm EPS is the two-member DNABII family of bacterial DNA-binding proteins, that includes histone-like protein (HU) and integration host factor (IHF) which are ubiquitous among all eubacteria (10). Importantly, HU and IHF lend essential structural support to the lattice-like network of eDNA in the biofilm EPS as they are positioned at the vertices of crossed strands of eDNA (10–12). Given the critical role DNABII proteins play in biofilm EPS structural integrity, we reasoned that they may prove to be a potentially effective and more universal target for development of a novel biofilm-disruption strategy. Indeed, we found that a monoclonal antibody directed against a synthetic ‘tip-chimer’ peptide immunogen, which we designed to mimic the immunoprotective DNA-binding ‘tips’ of a DNABII protein (‘α-DNABII’) (13), disrupts biofilms formed by 24 genera of bacterial pathogens tested to-date, and due to its specific mechanism of action, can also inhibit biofilm formation (14–18).

Moreover, we showed in four distinct pre-clinical models of human diseases wherein biofilms are known to contribute significantly to pathogenesis that α-DNABII treatment facilitates significant and rapid disease-resolution (19–22). Therapeutic treatment with α-DNABII facilitates clearance of aggregate *Pseudomonas aeruginosa* biofilms from the murine lung (20), resolves mucosal biofilms formed by *Aggregatibacter actinomycetemcomitans* in a rat model of osteolytic peri-implantitis (19), eradicates *Mycobacterium abscessus* from murine lungs with concomitant rapid return to homeostasis (22), and antigen-binding fragments derived from a humanized α-DNABII monoclonal resolved nontypeable *Haemophilus influenzae*-induced OM in the chinchilla (21). In terms of a preventive strategy, we’ve shown that active immunization with the tip-chimeric peptide prevented ascending experimental OM in chinchillas (23) and further, that we could use the tip-chimeric peptide as an active immunogen to redirect the natural acquired immune response in the chinchilla from one of preferential recognition of the non-protective tail domains to one of strong recognition of the protective tip domains without further amplification of the non-protective tail-directed response (24).

Whereas both our therapeutic monoclonal antibody-based approach and that of active immunization successfully facilitates disease resolution in multiple *in vivo* models (25), one question that continued to puzzle us was why the adaptive immune system does not generate similarly effective antibodies against these abundant DNABII proteins and thus naturally resolve biofilm-mediated diseases. We hypothesized that this may be due to the fact that when DNABII proteins are bound to eDNA, as they are within a biofilm matrix, the protective DNA-binding ‘tip’ domains of IHF or HU are physically occluded from immune processing. Through prior epitope-mapping studies we found this hypothesis to be correct, whereby eDNA-DNABII binding sterically occludes the immunoprotective DNA-binding tip regions of IHF and HU leaving only the non-protective ‘tail’ regions of a DNABII protein primarily exposed for immune response induction (13).

This new understanding led us to reason that it was thus highly likely that human sera would show a naturally skewed reactivity to the non-immunoprotective tail regions rather than to the immunoprotective tip regions of a DNABII protein, thereby necessitating the need to either provide the protective antibodies therapeutically or induce their formation by active immunization. In an earlier study of both children with active chronic OM as well as healthy adults, we found significantly greater recognition of the non-protective tail-chimeric peptide regardless of whether during periods of either active disease or health (24). Herein we endeavored to expand upon these early observations and specifically assess relative immune recognition of the DNABII epitopes now in those with additional well documented and extensively chronic biofilm-mediated disease, as well as healthy children.

## Methods

### Sex as a biological variable

Our studies involved both male and female children and adults and similar findings are reported for both sexes.

### Source of serum samples

For healthy children, sera were collected from children aged 1–4 yrs defined as having no evidence of otitis media (OM) or any other respiratory tract illness as indicated by their physician during a well-child visit, and were not currently taking antimicrobials (26) (**Table 1**). For OM-prone sera, these samples were recovered from children between the ages of 1–4 yrs that had experienced ≥ 4 episodes of acute OM in 1^st^ year of life or ≥ 6 episode by 2^nd^ year of life as documented by their pediatric otolaryngologist (26). Sera from adults with COPD aged 48–80 yrs were recovered from individuals followed in the COPD Study Clinic at the Buffalo VA Medical center at the time of exacerbation of disease as described (27, 28). Blood for serum was collected from PwCF aged 7–77 yrs during a routine clinic visit and under an approved IRB as detailed below.

**Table 1:** Demographic information of serum sample donors used in this study.

Serum sample origin	OM-prone children	Healthy children	People with COPD	People with cystic fibrosis
Age range (years)	1-4	1-4	48-80	7-77
Age, median (years)	1.5	2.5	69.5	22.5
Sex	62% male 38% female	44% male 56% female	100% male	40% male 60% female

### Blood sample collection

For use of sera isolated from fresh blood, whole blood was collected in red topped BD Vacutainer blood collection tubes without anti-coagulant (Becton Dickinson, Franklin Lakes, NJ) then allowed to sit at room temperature for 10 minutes until it clotted. Samples were then centrifuged at 2,000 g for 10 min at 4 °C followed by careful collection of the serum layer which was immediately frozen at -80 °C until use.

### Synthetic peptides and associated murine monoclonal antibodies

Murine monoclonal antibodies against the protective ‘tips’ of the DNABII proteins or non-protective ‘tails’ of the DNABII proteins (e.g., “MsTipMab” and “MsTailMab”) were purified from cell culture supernatants as previously described and validated (20). Peptide synthesis, purification, and sequence confirmation were performed by Ohio Peptide, LLC (for tip-chimeric peptide) and Genscript (for tail-chimeric peptide), and both chimeric peptides were confirmed to be ≥ 95% pure (24). As an additional control, a 40-mer peptide that mimicked a portion of an irrelevant outer membrane protein expressed by the same bacterium that was the origin of the DNABII proteins was integrated into the ELISA assay (29).

### Surface plasmon resonance

To first validate transition to an ELISA-based assay that mirrored surface plasmon resonance (SPR)-derived findings, a subset of sera from either healthy children or those with chronic OM were assessed by surface plasmon resonance (SPR) with a Biacore 3000 instrument (Cytiva, Marlborough, MA). A CM5 reagent grade sensor chip (Cytiva) was used to immobilize the tip- or tail-chimeric peptides to individual flow cells via amine coupling chemistry. A chip surface with no bound protein served as a negative control for these SPR assays. Once peptides bound, 15 µL of each serum was injected neat across the sensor chip surface at a flow rate of 5 μL serum/min, a sample volume and flow rate that achieved equilibrium. The surface was regenerated with 5 µL of NaOH 50 buffer (Cytiva). HBS-EP buffer (Cytiva) served as the running buffer.

### ELISA

**Figure 1:**
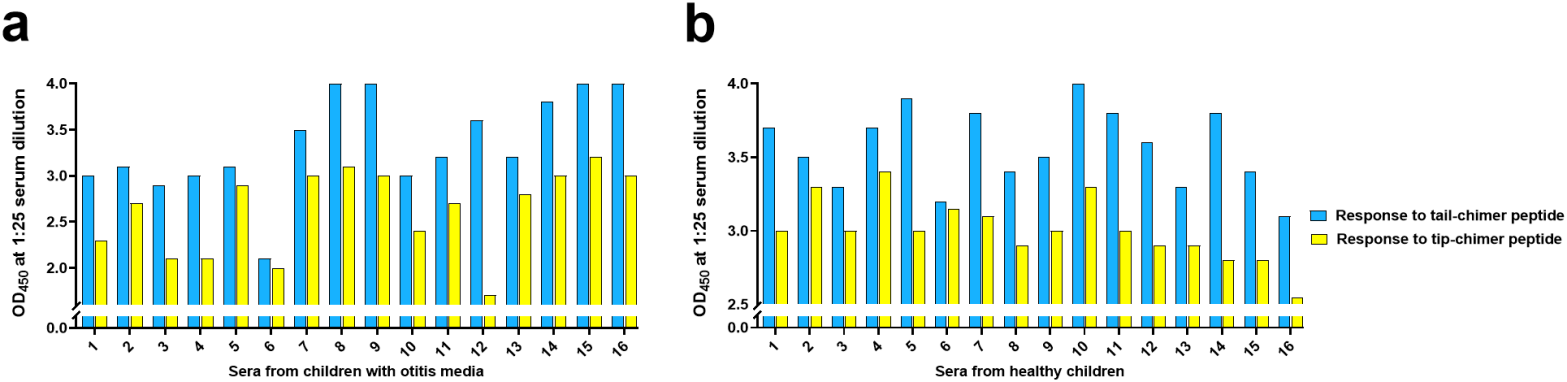
Children with chronic OM and healthy children exhibited adaptive IgG-specific immune responses skewed towards the non-immunoprotective tail domains of a DNABII protein. **(a)** In 15/16 (94%) samples assessed from children with chronic OM, the OD_450_ was significantly greater in response to the tail-chimeric peptide over the tip-chimeric peptide (p < 0.0001; OD_450_ ranges for on trend samples were: 1.7-3.1 against tip-chimeric peptide and 2.6-4.0 against tail-chimeric peptide). **(b)** Across 16 samples assessed from healthy children, the OD_450_ at a 1:25 dilution was significantly greater in 15/16 samples (94%) in response to the tail-chimeric peptide (p < 0.0001; OD_450_ ranges for on trend samples were: 2.6-3.4 against tip-chimeric peptide and 3.1-4.0 against tail-chimeric peptide).

**Figure 2:**
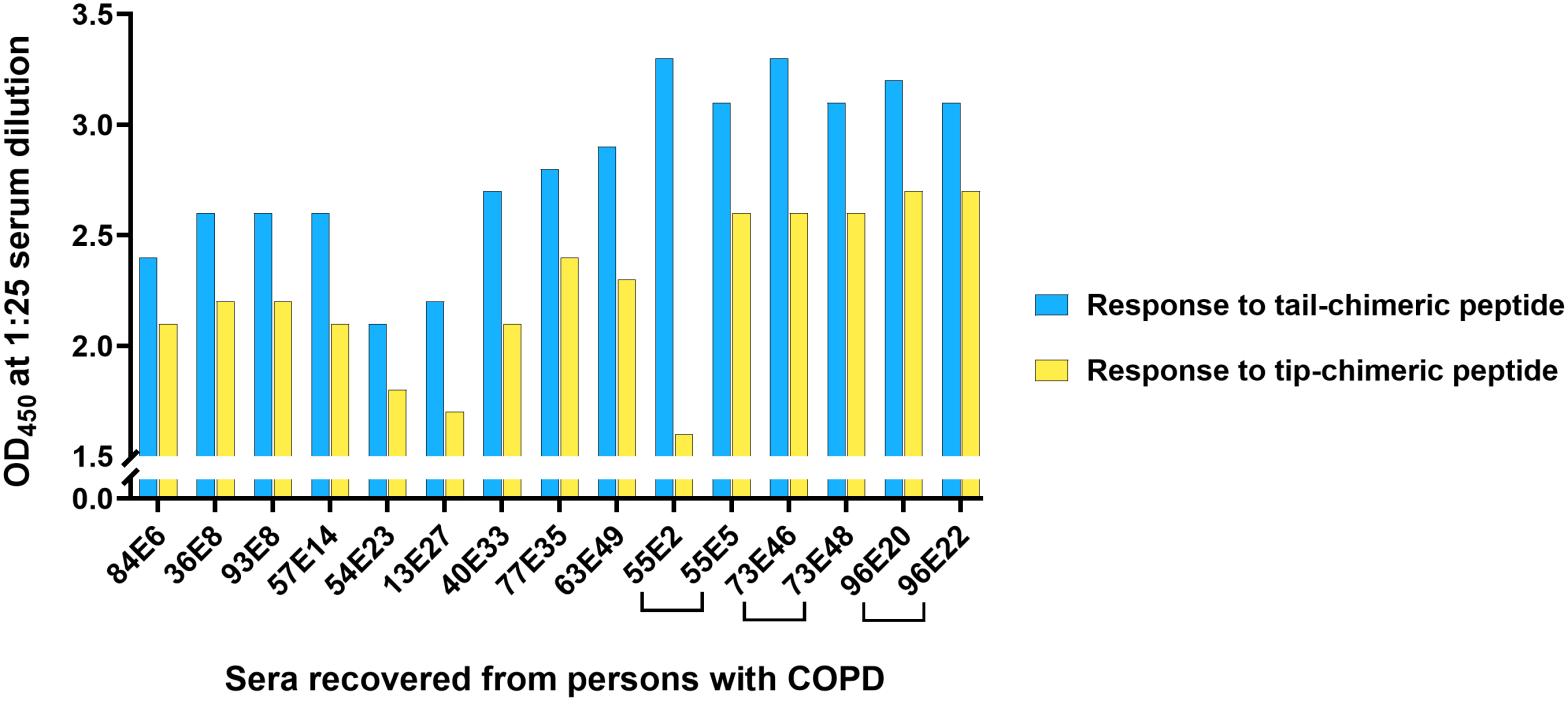
The natural IgG-specific adaptive immune response in people with chronic obstructive pulmonary disease was skewed towards the non-immunoprotective tails of a DNABII protein. In 15/15 (100%) samples from people with COPD, the reactivity to the tail-chimeric peptide was significantly greater than to the tip-chimeric peptide (p < 0.0001). Regardless of the number of exacerbations experienced (samples here represented a range of 6–49 exacerbations), immune recognition of the protective DNABII tip peptide never surpassed immunorecognition of the non-protective tail peptide. Further, dominant immunorecognition of the tail-chimeric peptide persisted within the same patient even as exacerbation number increased (see 3 individually bracketed samples). COPD samples are labeled as “#E#” where the first number represents the patient identifier number, “E” represents “exacerbation,” and the second number reflects the visit number of that patient in a prospective study. The OD_450_ ranges for sera from individuals with chronic COPD against tip-chimeric peptide was 1.6-2.7 and 2.1-3.3 against tail-chimeric peptide.

**Figure 3:**
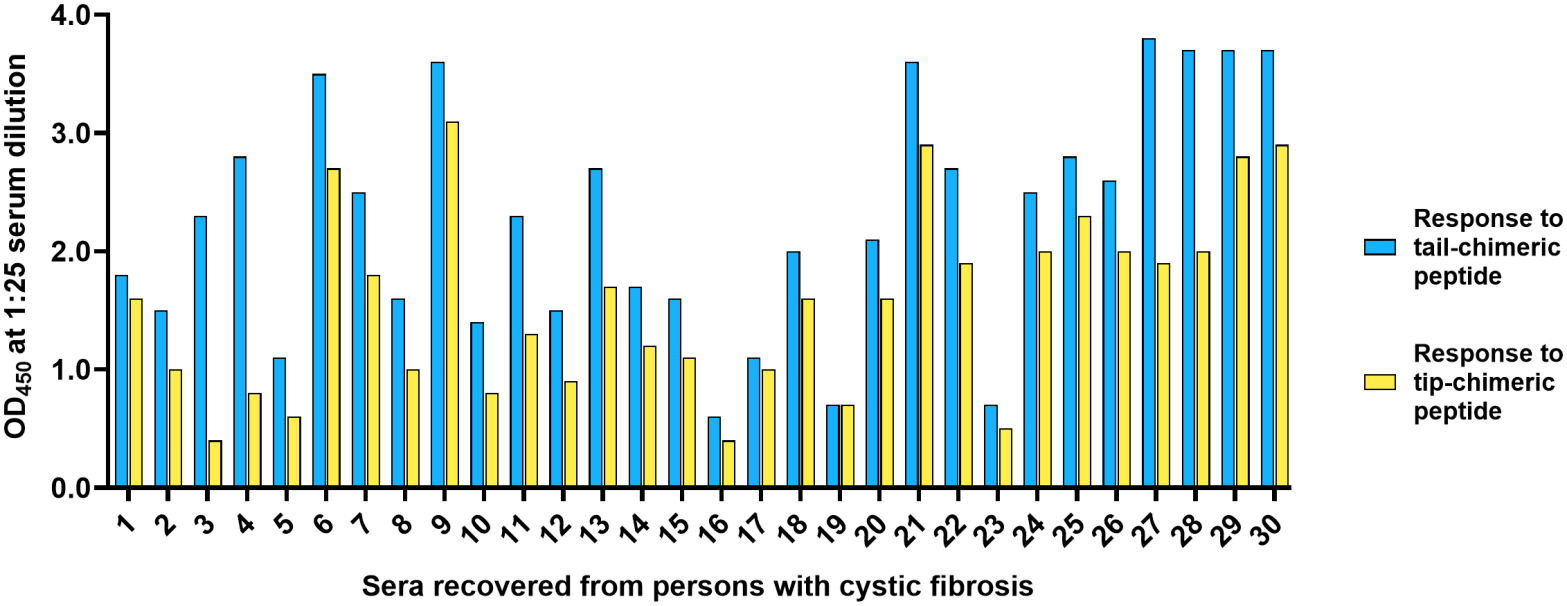
The natural IgG-specific adaptive immune response in fresh sera recovered from PwCF exhibited greater immunorecognition of the non-protective tail domain of a DNABII protein. In 29/30 (97%) freshly isolated serum samples recovered from a broad age range of PwCF, we found significantly skewed and preferential recognition of the non-protective tail- over the protective tip-chimeric peptide (p < 0.0001). Sample #19 was the sole example of equivalent recognition of both peptides. The OD_450_ ranges for on trend samples were 0.4-3.1 against the tip-chimeric peptide and 0.6-3.8 against the tail-chimeric peptide.

To assess preferential binding of antibodies within either archived or fresh serum samples fromchildren with chronic OM, healthy children, children and adults with CF or adults with COPD to either tip- or tail-chimeric peptides, an enzyme-linked immunosorbent assay was developed. Either 0.05 μg tip-chimeric peptide/50 μL carbonate-bicarbonate buffer (pH 9.6) or 0.2 μg of either an irrelevant 40-mer peptide or the tail-chimeric peptide/50 μL carbonate-bicarbonate buffer was added to each well of MaxiSorp C-bottom microtiter plates (Thermo Fisher Scientific, Waltham, MA) and adsorbed overnight at 4 °C. Wells were then blocked with 2% skim milk in 10 mM phosphate buffered saline (pH 7.4; PBS). After 1 h incubation at 4 °C, wells were washed with 10 mM PBS, followed by addition of either 0.1 μg MsTipMab/50 μL 10 mM PBS or 1.5 μg MsTailMab/50 μL 10 mM PBS per well, or dilution of each serum sample (1:10, 1:25, 1:50, 1:100, 1:125, and 1:150) per well. Due to natural differences between monoclonal antibodies, concentrations of chimeric peptides and their respective monoclonal antibody were chosen such that the resulting OD_450_ in the positive control wells exhibited an equivalent and similarly strong signal (**Supplementary Figure 1**). We intentionally aimed for a maximal readout of 4.0 for positive controls in order to be able to readily detect any potential problematic cross-reactivity; none was detected as evidenced by values in negative control wells. After 1 h incubation, wells were washed as before and Protein A conjugated to horseradish peroxidase (Invitrogen, Waltham, MA) was added to predominantly detect IgG1/2/4 as human IgG3 binds poorly to Protein A and IgM/IgA are typically weakly detected. Color was developed with One-Step Ultra TMB (Thermo Fisher Scientific), and the reaction stopped with 2 M H_2_SO_4_ after approximately 5 minutes. The resulting OD_450_ was read via plate reader (FLUOstar Omega) for all six dilutions of sera. Due to the limited volume of pediatric sera, each sample was run once and values of OD_450_ obtained at a 1:25 dilution of each serum sample are reported in **Figures 1**-**3** in order to maintain a single standard for all tested sera (see **Supplementary Figures 7–9** for trends across dilutions).

### Statistics

Paired parametric t-tests were performed for all normally distributed surface plasmon resonance and ELISA optical density data to detect relative differences in recognition of tip- versus tail-chimeric peptides. For datasets not normally distributed, paired nonparametric t-tests were performed. Due to the small sample sizes used throughout the manuscript, a Shapiro-Wilk test for normality was performed on all data to ensure that any stated statistical significance with parametric t-tests were retained and all Shapiro-Wilk normality tests are graphically presented (**Supplementary Figure 2**). To determine correlation between SPR and ELISA datasets, Spearman correlation tests were performed and presented (**Supplementary Figure 3**). Results from parametric testing are retained when data were normally distributed. All statistical analyses were performed in GraphPad Prism (v. 10.3.1).

### Study approval

De-identified human blood donations were made under the auspices of the Cure Cystic Fibrosis Columbus (C3) Research and Development Program at Nationwide Children’s Hospital after informed written consent was obtained. Collection of all de-identified blood was performed under protocols approved by the Nationwide Children’s Hospital Institutional Review Board (protocols #0312HS196, #IRB17-00968). De-identified serum samples from those with COPD were similarly collected after obtaining written informed consent under protocols approved by the Institutional Review Boards of the University at Buffalo and the Western New York Veterans Affairs Healthcare System. COPD samples are labeled as “#E#” where the first number represents the patient number, and the second number reflects the visit number of that patient when they were experiencing an exacerbation of disease during a prospective study. Use of whole blood and isolated sera were approved for use in studies conducted within our laboratory in conformity with, and as approved under, Nationwide Children’s Hospital Institutional Biosafety Committee (IBC) protocol #IBS-00000449.

## Results

### IgG antibodies in sera from children with chronic otitis media exhibited greater reactivity to the non-protective domains of DNABII proteins

We first confirmed the integrity of 10 archived frozen sera recovered from children with chronic OM and found both strong analyte-ligand binding kinetics, and reproducible immunoreactivity to that shown in earlier work (24) (**Supplementary Figures 4a-j**). Across ten samples from children with chronic OM, nine (90%) exhibited significantly greater response to the tail-chimeric peptide (blue sensorgrams; mean resonance units ± SEM: 698 ± 39) than to the tip-chimeric peptide (yellow sensorgrams; mean RU ± SEM: 231 ± 24) (p = 0.004). Only 1 of 10 samples (**Supplementary Figure 4j**; 10%) exhibited similar recognition of both chimeric peptides although that to the tail peptide was nonetheless slightly greater.

Six randomly chosen of these same samples (including both typically tip-skewed representatives of the trend as well as sample ‘j’) were then re-assessed via a newly developed ELISA. We found a high degree of similarity between trends obtained within our SPR dataset and those generated via ELISA, wherein reactivity to the tail-chimeric peptide (blue bars) was greater than reactivity to the tip-chimeric peptide (yellow bars) in 6/6 (100%) of these pediatric samples, with that of sample ‘j’ showing the least difference, all of which were highly similar to SPR results [**Figures 1a**, samples #1-6 (#6 is sample ‘j’)].

With confirmation that our ELISA-based results aligned well with those obtained via SPR (**Supplementary Figures 3 a, b**, note r values of 0.99 or 0.97 and associated p-values of 0.006 & 0.01, respectively), we proceeded to assess ten additional archived frozen serum samples from children with OM (**Figure 1a**, samples #7-16). We found that 10/10 (100%) samples exhibited significantly greater recognition of the tail-chimeric peptide (blue bars) compared to recognition of the tip-chimeric peptide (yellow bars) (p = 0.0002). Additionally, the combined responses to either chimeric peptide by all 16 samples from OM-prone children are also presented (**Supplementary Figure 5**).

### The natural IgG-specific adaptive immune response in healthy children was also skewed towards the non-protective domains of DNABII proteins

Assessment of 10 archived frozen healthy pediatric serum samples for integrity via SPR also demonstrated strong and consistent analyte-ligand binding kinetics (**Supplementary Figures 6a-j**). Out of ten serum samples assessed from healthy children, nine (90%) exhibited significantly greater reactivity to the tail-chimeric peptide (blue sensorgrams; mean RU ± SEM: 373 ± 51) relative to response to the tip-chimeric peptide (yellow sensorgrams; mean RU ± SEM: 143 ± 32) (p = 0.009). Serum from one child showed equivalent recognition of both peptides (**Supplementary Figure 6h**). Interestingly, we found that healthy children also showed preferential recognition of the tail-chimeric peptide although the magnitude of this recognition was overall less than that of sera recovered during active OM (e.g., 373 ± 51 for healthy children compared to 698 ± 39 for children with chronic OM). For reference, mean RU values for sera from healthy adults were 616 to the tail-chimeric peptide versus 253 to the tip-chimeric peptide (24). When 6 of these 10 samples from healthy children (including sample ‘h’ which broke the trend) were then tested via ELISA, preferential recognition of the non-protective tail was again observed with the exception of the sample which broke the trend, which again overall aligned well with data generated by SPR with only the response of healthy children to the tip chimer showing a non-significant correlation despite an r value of 0.76 once the trend breaking sample was incorporated [**Figure 1b**, samples #1-6 (#6 is sample ‘h’)] (**Supplementary Figures 3b, c** r values of 0.76 & 0.99 with associated p-values of 0.12 & 0.008, respectively). As such, we proceeded to assess reactivity in ten additional healthy pediatric sera and found that 10/10 (100%) of these additional samples exhibited similarly significantly greater reactivity to the tail- over the tip-chimeric peptide (**Figure 1b**, samples #7-16) (p = 0.002). Combined responses to either chimeric peptide by all 16 serum samples from healthy children are also presented (**Supplementary Figure 5**).

### The natural IgG-specific adaptive immune response in people with COPD was skewed towards the non-immunoprotective tail domains of a DNABII protein

With correlation between SPR and ELISA data validated (**Supplementary Figure 3**), we proceeded to assess potential preferential immunoreactivity in 15 archived frozen serum samples recovered from adults experiencing an exacerbation of COPD. Of 15 total sera assessed, 15/15 samples (100%) exhibited significantly greater reactivity to the tail-chimeric peptide than the tip-chimeric peptide (p < 0.0001) (**Figure 2**). Interestingly, regardless of additional experienced exacerbations, as demonstrated in three paired samples from patients #55, 73 or 96, immune recognition did not appear to shift in favor of tip-chimeric peptide recognition (**Figure 2**, bracketed sample pairs). Further, samples tested herein represent a range of 6–49 total exacerbations experienced by any given individual with COPD, yet regardless of the number of exacerbations experienced, the immune recognition of the non-protective tail region consistently surpassed immune recognition of the protective DNABII tip region. It is important to note that the COPD cohort is highly gender skewed (e.g. 100% male) and thereby a potential limitation to the generalizability of our data, however it is important to note that each patient is compared only to themselves.

### The natural IgG-specific adaptive immune response in PwCF exhibited greater reactivity to the non-protective domains of DNABII proteins

In addition to archived frozen serum samples, we also wanted to assess fresh samples from another group who experience recalcitrant bacterial biofilm-mediated lung infections, people with cystic fibrosis (PwCF). As such we evaluated 30 freshly recovered sera from PwCF aged 7–77 years and found that 29 samples (97%) exhibited significantly greater reactivity to the tail- over tip-chimeric peptide, an observation that was consistent across the tested age span (p < 0.0001) (**Figure 3**). Only one sample, #19, exhibited similar recognition of both peptides via ELISA.

## Discussion

We previously showed that the structural biofilm eDNA matrix is stabilized by a common component: the bacterial nucleoid-associated family of DNABII proteins, IHF and HU (13). Additionally, through epitope mapping efforts, we revealed that the immuno-protective ‘tip’ domains of the DNABII proteins, when bound to eDNA, are occluded from the host immune system, leaving primarily the non-protective ‘tail’ domains exposed for generation of non-protective antibodies (24, 30). In early SPR-based analysis of serum from 25 children with otitis media and 10 healthy adults, we showed for the first time that the adaptive immune response is indeed skewed toward the DNABII protein’s nonprotective tails (24). In the same published work, we showed that similar skewing was evident in sera from healthy chinchillas wherein the response to immunization with a native IHF protein only further augments the highly preferentially skewed immune response to the nonprotective tail domains (24). Conversely, however, when we specifically immunized a chinchilla with the tip-chimeric immunogen, the adaptive immune response was now strongly skewed toward the immuno-protective tip domains without any further augmentation of the non-protective tail-directed response.

Here, we bridged to our preliminary SPR-based work (24) to now more fully examine the preferential immune responsiveness of children with chronic OM, as well as healthy children who surely have healthy biofilms as well as likely a history of prior biofilm-mediated disease, to the DNABII proteins and showed, in both groups, that a non-protective response dominated. Whereas this non-protective response would likely spare those healthy commensal biofilms with which we have co-evolved for their many positive benefits (31, 32), it also fails to eradicate pathogenic biofilms. Additionally, to expand upon our early observations, herein we showed that this skewed adaptive immune response was also operational in adults with COPD and in children and adults with an additional years-long biofilm-mediated infections due to CF. Further, we showed in those with COPD, that the immune system failed to shift towards a protective tip-specific response despite repeated exacerbations of disease. Within the compiled data of sera from PwCF there was a single outlier wherein recognition of both the tip- and tail-chimeric peptides was equivalent. We are unable to state with full confidence &as to why any of the 3 samples described herein broke the trend, as we did not have access to patient histories to accompany de-identified serum samples, however given the ‘snapshot in time’ nature of this evaluation of a single serum sample from each participant there are likely multiple potential underlying reasons. As such we could speculate that given that these samples which broke the trend tended to have lower overall responses, this could be reflective of individuals who are typically generally low responders, however it may simply reflect a point in either disease development or resolution wherein a skewed response had not yet developed. It would seem less likely that a previously tail-skewed response had resolved given that these trend breaking responses were both rare herein as well as not detected at all in an earlier published study (24). Further, in sera recovered from individuals with COPD as tested here, the tail-skewed response did not transition to a tip-skewed response even with multiple disease exacerbations. Nonetheless, given that the remaining sera evaluated all demonstrated the characterized and clearly skewed adaptive immune recognition of the non-protective domains of DNABII proteins in each of these groups, it appears likely that this outcome contributes notably to our inability to naturally resolve chronic and recurrent biofilm mediated diseases. It is important to note that due to the fact that we used Protein-A-HRP for our ELISA readouts, data presented herein predominantly reflect detection of IgG1/2/4. Thereby, our results and conclusions drawn are restricted to the IgG-specific adaptive immune responsiveness of the individuals whose sera were tested herein.

Collectively, whereas the data presented both here and in earlier work (24) do not provide definitive proof as to the exact mechanism, they nonetheless are consistent with the concepts of original antigenic sin (33, 34), wherein we hypothesize that our initial response to either a healthy biofilm or to a biofilm-mediated infection induces the formation of a non-protective tail-specific response which becomes only further exacerbated by subsequent infections due to the orientation in which DNABII proteins are embedded in the eDNA-rich biofilm matrix (34). For this reason, active immunization with the native DNABII protein would likely also only further promote the induced tail-dominant immune response. Thus, we developed the DNABII tip-chimeric peptide-based vaccine candidate which is designed to specifically redirect the immune response to one of immunoprotection, ultimately inducing the collapse of biofilms in a what appears to date to be a species agnostic manner to mediate rapid disease resolution as we have shown occurs pre-clinically (24, 25).

To attempt to overcome this immunological obstacle, we developed two approaches. The first involves delivery of a humanized monoclonal to provide the needed immunoprotective antibodies to those with chronic and/or recurrent bacterial disease as a therapeutic approach which is currently in first-in-human clinical trials (NCT05629741; NCT06159725). The second approach is one of active immunization with the tip-chimeric peptide used as a vaccine candidate to redirect the immune system during disease and thereby induce the formation of antibodies directed towards the protective DNABII tip domains. For each of these two approaches to be highly effective, they must not induce collateral damage to any healthy biofilms. We are encouraged by the observation that in preclinical models, no dysbiosis of the gut or nasopharyngeal flora have been demonstrated (31, 32), however we are, and will continue to, actively monitoring for any off-target effects during clinical evaluations.

As biofilm-mediated infections readily evade clearance by both traditional antibiotics and the host’s immune system, novel disease resolution strategies are urgently needed. In new work presented here, we provide additional rationale for an immunization strategy that targets a ubiquitous and essential structural biofilm EPS component, the DNA-binding bacterial DNABII proteins. The relatively small sample sizes for the evaluated sera is a limitation of the study as is the fact that we do not have access to the complete health records of any participant whose sera we tested. Nonetheless, these new findings suggest the need for redirection of the host natural immune response toward the masked epitopes of the DNABII protein that lend biofilms their stability. This redirection could potentially empower the host adaptive immune system to rapidly eradicate these recalcitrant biofilms and resolve disease. Validation of this latter hypothesis will require clinical trials, a goal we are currently working towards.

## Data Availability

The original contributions presented in the study are included in the article/**Supplementary Material**. Further inquiries can be directed to the corresponding author.

## Supplementary material

Supplementary Figure 1 Representative image of ELISA control wells. Via our newly developed enzyme linked-immunosorbent assay, designed due to its greater accessibility within laboratories, we ensured our monoclonal antibodies directed against either a synthetic tip- or tail-chimeric peptide demonstrated equivalent recognition of their targets. As evidenced from the dark blue hue in positive control wells (with OD_450_ values of 4.0 which represented the maximum value of the plate reader as intentionally used in order to better detect any cross-reactivity or false positives) *versus* the very light blue-white hues in all negative control wells, we confirmed both the equivalency and specificity of immunorecognition of each chimeric peptide.

Supplementary Figure 2 Results of Shapiro-Wilk normality tests for SPR and ELISA datasets. Normality tests were performed to assess normality of SPR and ELISA datasets. Normality for SPR data from serum samples from OM-prone children (a) and healthy children (b) exhibited non-normal distribution and normal distribution, respectively. ELISA data from OM-prone children (c), healthy children (d), people with COPD (e), and people with CF (f) were all normally distributed. For datasets that were normally distributed, a parametric paired t-test was performed to assess statistical significance. For datasets that were not normally distributed, a nonparametric paired t-test was performed to account for the non-normality and relatively small sample size.

Supplementary Figure 3 Spearman correlation between ELISA and SPR datasets to ensure differing methodologies aligned. To ensure that data obtained via our newly developed ELISA and our SPR datasets aligned, we assessed correlation between data obtained via ELISA and SPR in reactivity to the tail- or tip-chimeric peptides in five randomly chosen serum samples from OM-prone (panels a & b) or healthy (panels c & d) children via nonparametric Spearman correlation. In all cases, we found a very high degree of correlation (r = 0.76-0.99) between results obtained via SPR versus ELISA, which provided support for the validity of transitioning from an SPR-based methodology to the more widely accessible ELISA methodology. Note: some symbols were changed from open circles to open triangles in panel b to allow better resolution of the 5 ‘on trend’ data points that cluster together away from the rare single outlier data point.

Supplementary Figure 4 Via surface plasmon resonance, archived frozen sera from children with otitis media exhibited a preferentially skewed response towards the tail-chimeric peptide. Nine of ten serum samples (90%) recovered from children with chronic OM, exhibited significantly (p < 0.01) greater recognition of the tail-chimeric peptide (blue sensorgrams; mean RU ± SEM: 698 ± 39) compared to recognition of the tip-chimeric peptide (yellow sensorgrams; mean RU ± SEM: 231 ± 24). One serum sample (see sample in panel j) exhibited recognition to the tail-chimeric peptide that while greater than to the tip-chimeric peptide (e.g. 206 vs. 134, respectively), this difference was not significant.

Supplementary Figure 5 Bar and scatter plots of sera sample reactivities to the tail- or tip-chimeric peptide via ELISA. All reactivities to either chimeric peptide in serum samples from OM-prone children (a), healthy children (b), people with COPD (c), or people with CF (d) are presented to aid visualization of the spread and distribution for each group of assessed individuals. Statistical significance was indicated for all serum sample sets (p < 0.0001).

Supplementary Figure 6 Via surface plasmon resonance, archived frozen sera from healthy children exhibited a preferentially skewed response towards the tail-chimeric peptide. Of ten serum samples assessed from healthy children, 9 (90%) exhibited significantly (p < 0.01) greater response to the tail-chimeric peptide (blue sensorgrams; mean RU ± SEM: 373 ± 51) relative to response to the tip-chimeric peptide (yellow sensorgrams; mean RU ± SEM: 143 ± 32). For one child (see panel h) the responses were equivalent to the tail- and tip-chimeric peptides (e.g. 251 vs. 261 RU, respectively). Overall, however, regardless of whether sera originated from children with chronic OM or healthy children, immunorecognition of the non-protective domain of a DNABII protein exceeded that of the protective domain.

Supplementary Figure 79 Plots depict trends in ELISA data that are maintained over the dilution series tested. **Supplementary Figure 7a** – otitis media prone children and Panel b – healthy children as shown in **Figure 1**, respectively.

Supplementary Figure 8 – People with COPD as shown in **Figure 2**.

Supplementary Figure 9 – People with Cystic Fibrosis as shown in **Figure 3**.
