# Supplementary material for: The natural human adaptive IgG-specific immune response is skewed towards non-protective tail domains of DNABII proteins
Source: Front Immunol. 2026 Feb 27;17:1694547. doi: 10.3389/fimmu.2026.1694547 (PMC12982335; doi:10.3389/fimmu.2026.1694547)
Supplement: Supplementary Figure 1 — Representative image of ELISA control wells. Via our newly developed enzyme linked-immunosorbent assay, designed due to its greater accessibility within laboratories, we ensured our monoclonal antibodies directed against either a synthetic tip- or tail-chimeric peptide demonstrated equivalent recognition of their targets. As evidenced from the dark blue hue in positive control wells (with OD450 values of 4.0 which represented the maximum value of the plate reader as intentionally used in order to better detect any cross-reactivity or false positives) versus the very light blue-white hues in all negative control wells, we confirmed both the equivalency and specificity of immunorecognition of each chimeric peptide. [file DataSheet1.pdf]

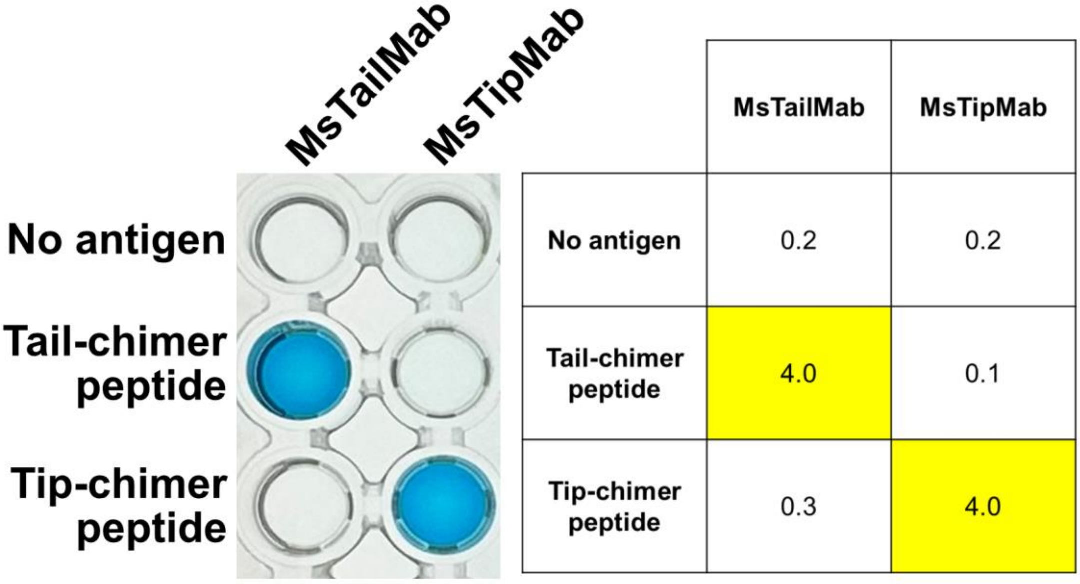

	MsTailMab	MsTipMab
No antigen	0.2	0.2
Tail-chimer peptide	4.0	0.1
Tip-chimer peptide	0.3	4.0

Supplementary Figure 1. Representative image of ELISA control wells
Via our newly developed enzyme linked-immunosorbent assay, designed due to its greater accessibility within laboratories, we ensured our monoclonal antibodies directed against either a synthetic tip- or tail-chimeric peptide demonstrated equivalent recognition of their targets. As evidenced from the dark blue hue in positive control wells (with OD_{450} values of 4.0 which represented the maximum value of the plate reader as intentionally used in order to better detect any cross-reactivity or false positives) *versus* the very light blue-white hues in all negative control wells, we confirmed both the equivalency and specificity of immunorecognition of each chimeric peptide.

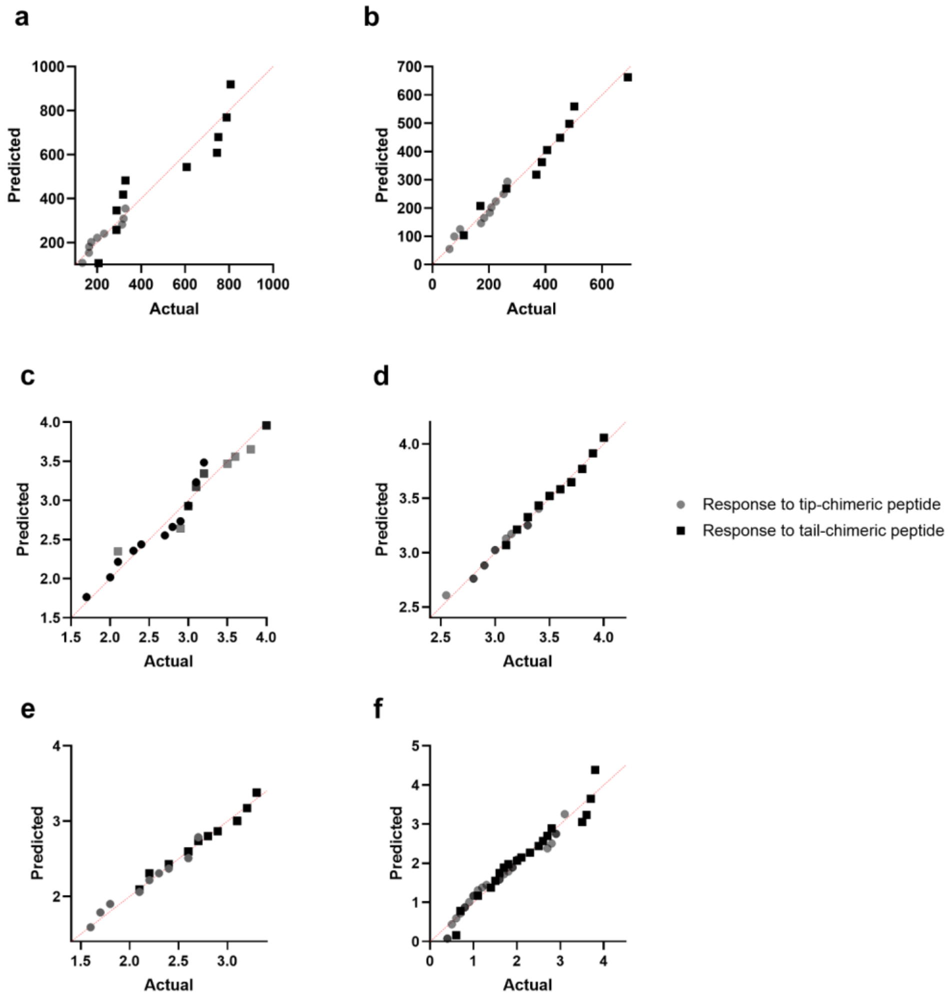

Supplementary Figure 2. Results of Shapiro-Wilk normality tests for SPR and ELISA datasets

Normality tests were performed to assess normality of SPR and ELISA datasets. Normality for SPR data from serum samples from OM-prone children (a) and healthy children (b) exhibited non-normal distribution and normal distribution, respectively. ELISA data from OM-prone children (c), healthy children (d), people with COPD (e), and people with CF (f) were all normally distributed. For datasets that were normally distributed, a parametric paired t-test was performed to assess statistical significance. For datasets that were not normally distributed, a nonparametric paired t-test was performed to account for the non-normality and relatively small sample size.

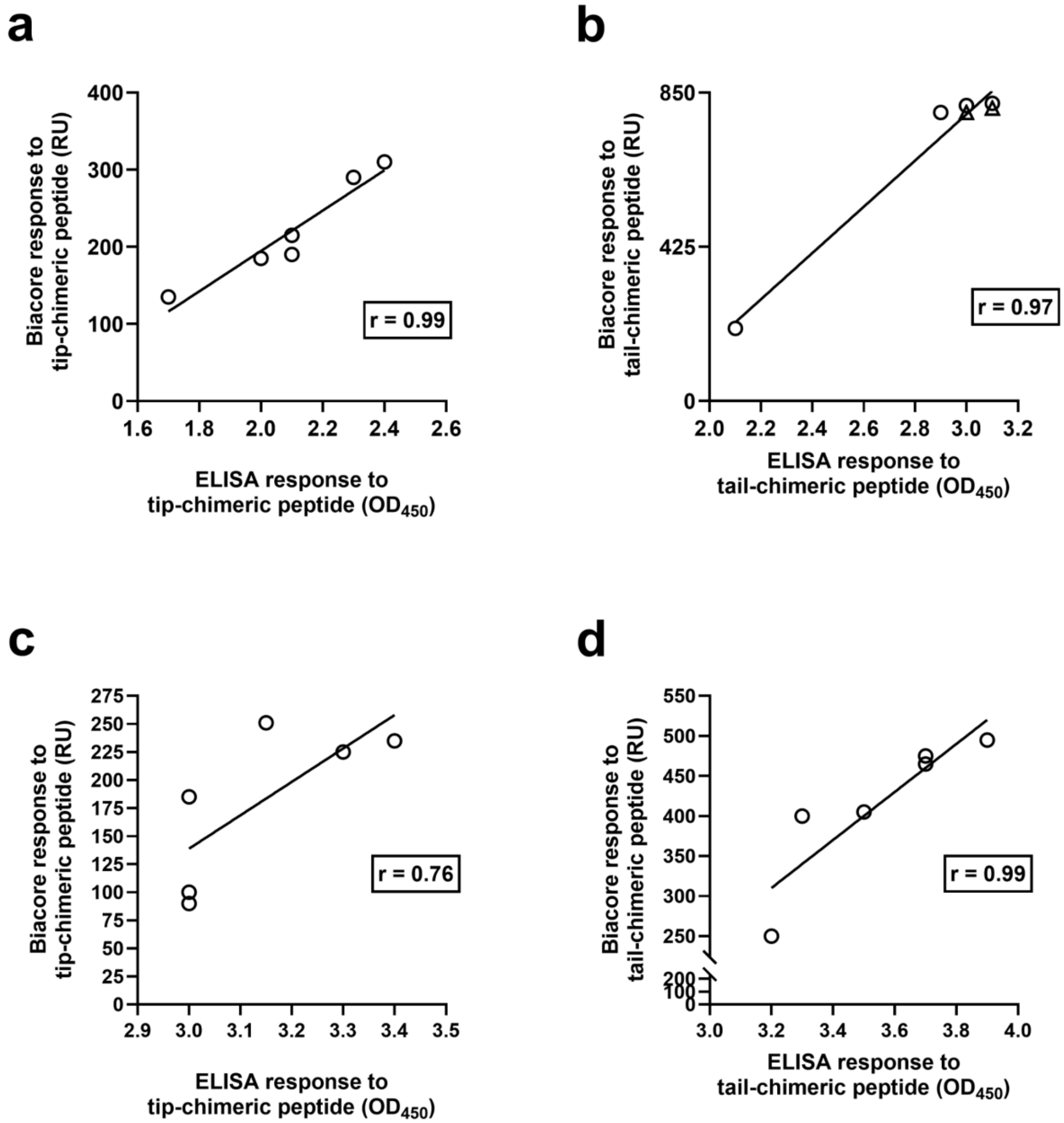

Supplementary Figure 3. Spearman correlation between ELISA and SPR datasets to ensure differing methodologies aligned

To ensure that data obtained via our newly developed ELISA and our SPR datasets aligned, we assessed correlation between data obtained via ELISA and SPR in reactivity to the tail- or tip-chimeric peptides in five randomly chosen serum samples from OM-prone (panels a & b) or healthy (panels c & d) children via nonparametric Spearman correlation. In all cases, we found a very high degree of correlation (r = 0.76-0.99) between results obtained via SPR versus ELISA, which provided support for the validity of transitioning from an SPR-based methodology to the more widely accessible ELISA methodology. Note: some symbols were changed from open circles to open triangles in panel b to allow better resolution of the 5 ‘on trend’ data points that cluster together away from the rare single outlier data point.

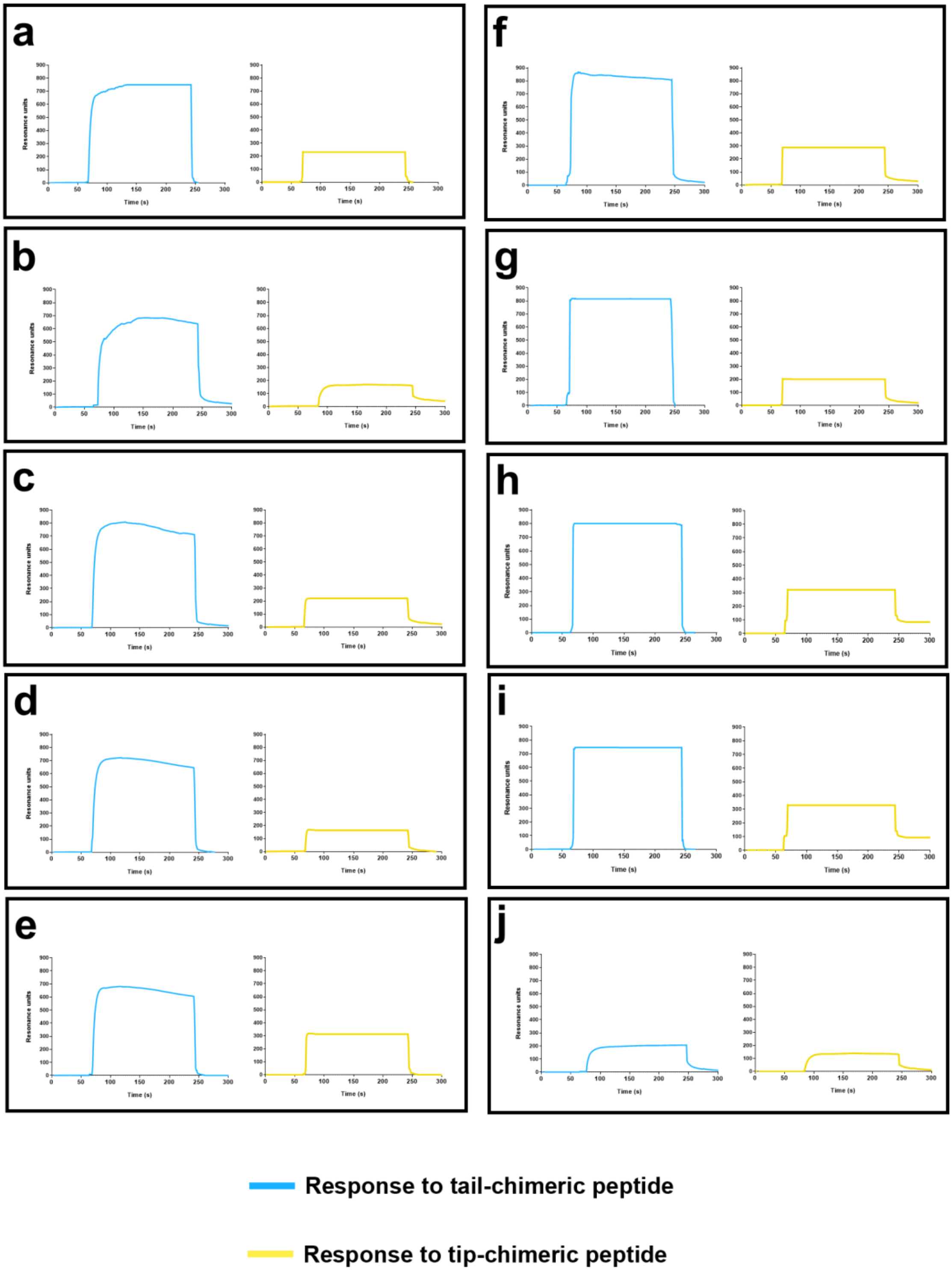

Supplementary Figure 4. Via surface plasmon resonance, archived frozen sera from children with otitis media exhibited a preferentially skewed response towards the tail-chimer peptide

Nine of ten serum samples (90%) recovered from children with chronic OM, exhibited significantly ($p < 0.01$) greater recognition of the tail-chimeric peptide (blue sensorgrams; mean RU ± SEM: 698 ± 39) compared to recognition of the tip-chimeric peptide (yellow sensorgrams; mean RU ± SEM: 231 ± 24). One serum sample (see sample in panel j) exhibited recognition to the tail-chimeric peptide that while greater than to the tip-chimeric peptide (e.g., 206 vs. 134, respectively), this difference was not significant.

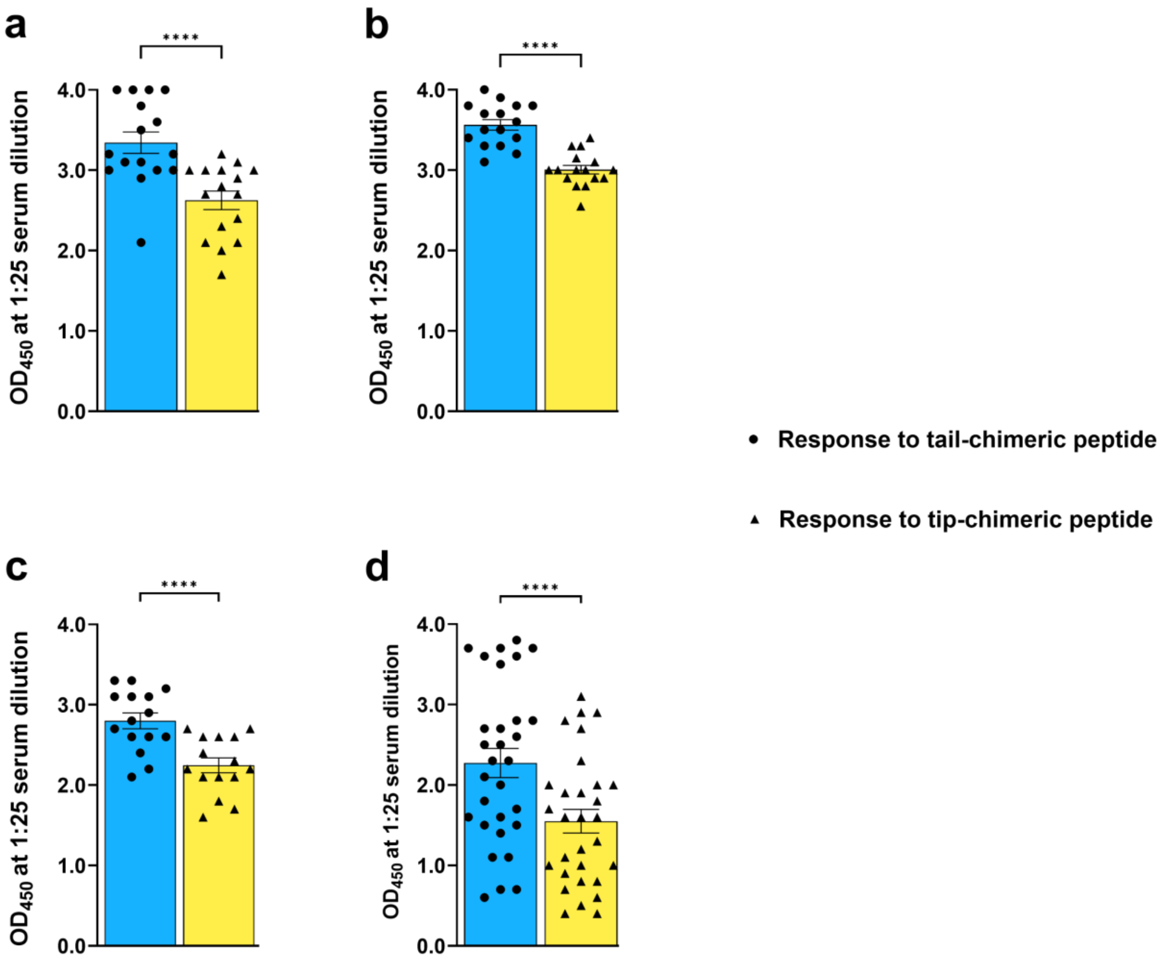

Supplementary Figure 5. Bar and scatter plots of sera sample reactivities to the tail- or tip-chimeric peptide via ELISA

All reactivities to either chimeric peptide in serum samples from OM-prone children (a), healthy children (b), people with COPD (c), or people with CF (d) are presented to aid visualization of the spread and distribution for each group of assessed individuals. Statistical significance was indicated for all serum sample sets ($p < 0.0001$).

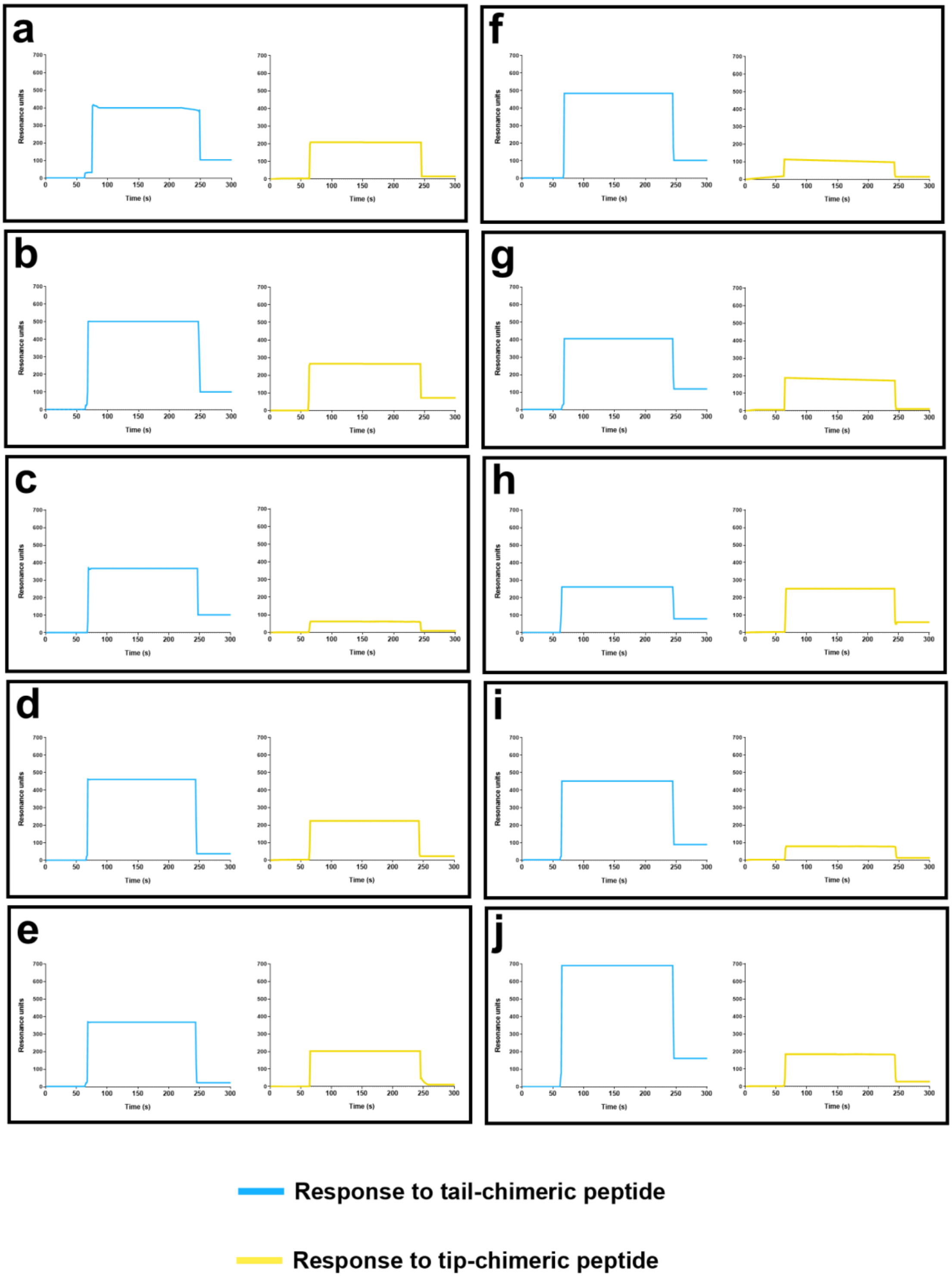

Supplementary Figure 6. Via surface plasmon resonance, archived frozen sera from healthy children exhibited a preferentially skewed response towards the tail-chimer peptide
Of ten serum samples assessed from healthy children, 9 (90%) exhibited significantly ($p < 0.01$) greater response to the tail-chimeric peptide (blue sensorgrams; mean RU ± SEM: 373 ± 51) relative to response to the tip-chimeric peptide (yellow sensorgrams; mean RU ± SEM: 143 ± 32). For one child (see panel h) the responses were equivalent to the tail- and tip-chimeric peptides (e.g., 251 vs. 261 RU, respectively). Overall, however, regardless of whether sera originated from children with chronic OM or healthy children, immunorecognition of the non-protective domain of a DNABII protein exceeded that of the protective domain.

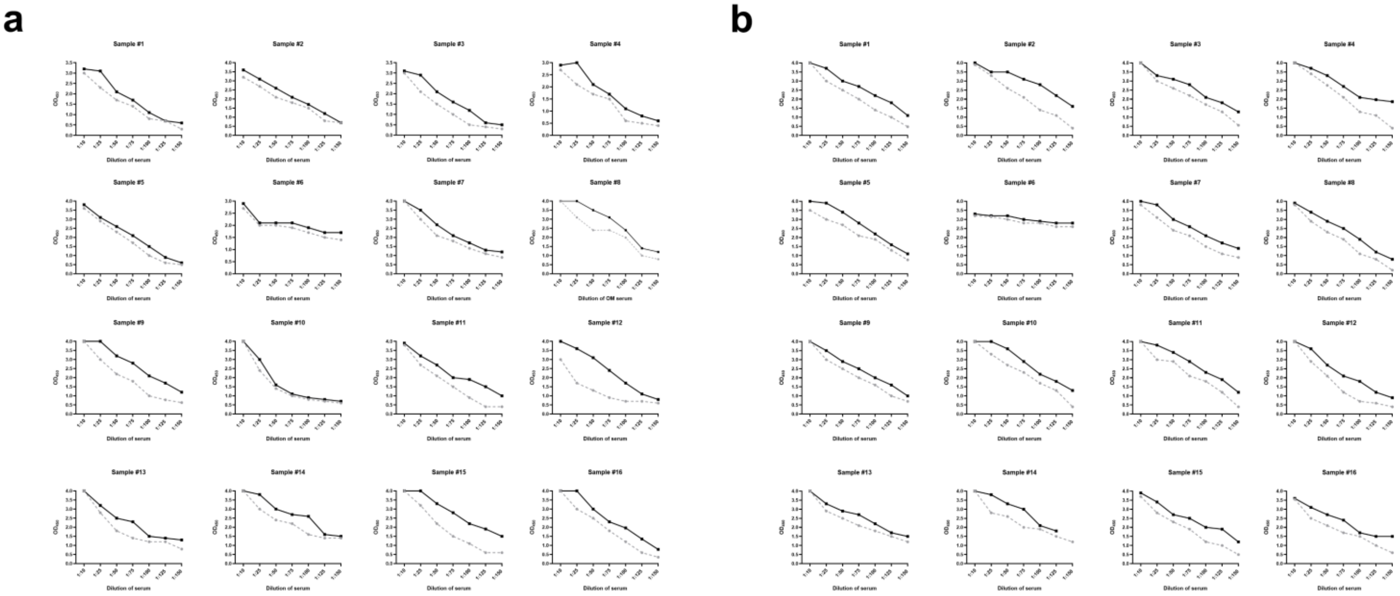

Supplementary Figure 7. Plots depict trends in ELISA data that are maintained over the dilution series tested

Panel a – otitis media prone children and Panel b – healthy children as shown in Figure 1, respectively.

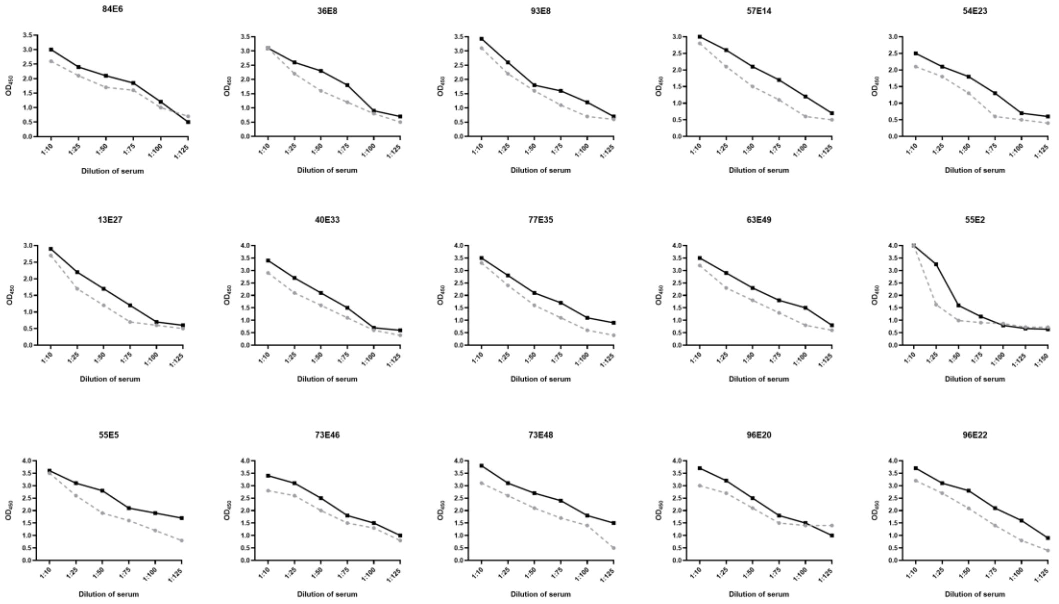

Supplementary Figure 8. Plots depict trends in ELISA data that are maintained over the dilution series tested

People with COPD as shown in Fig. 2.

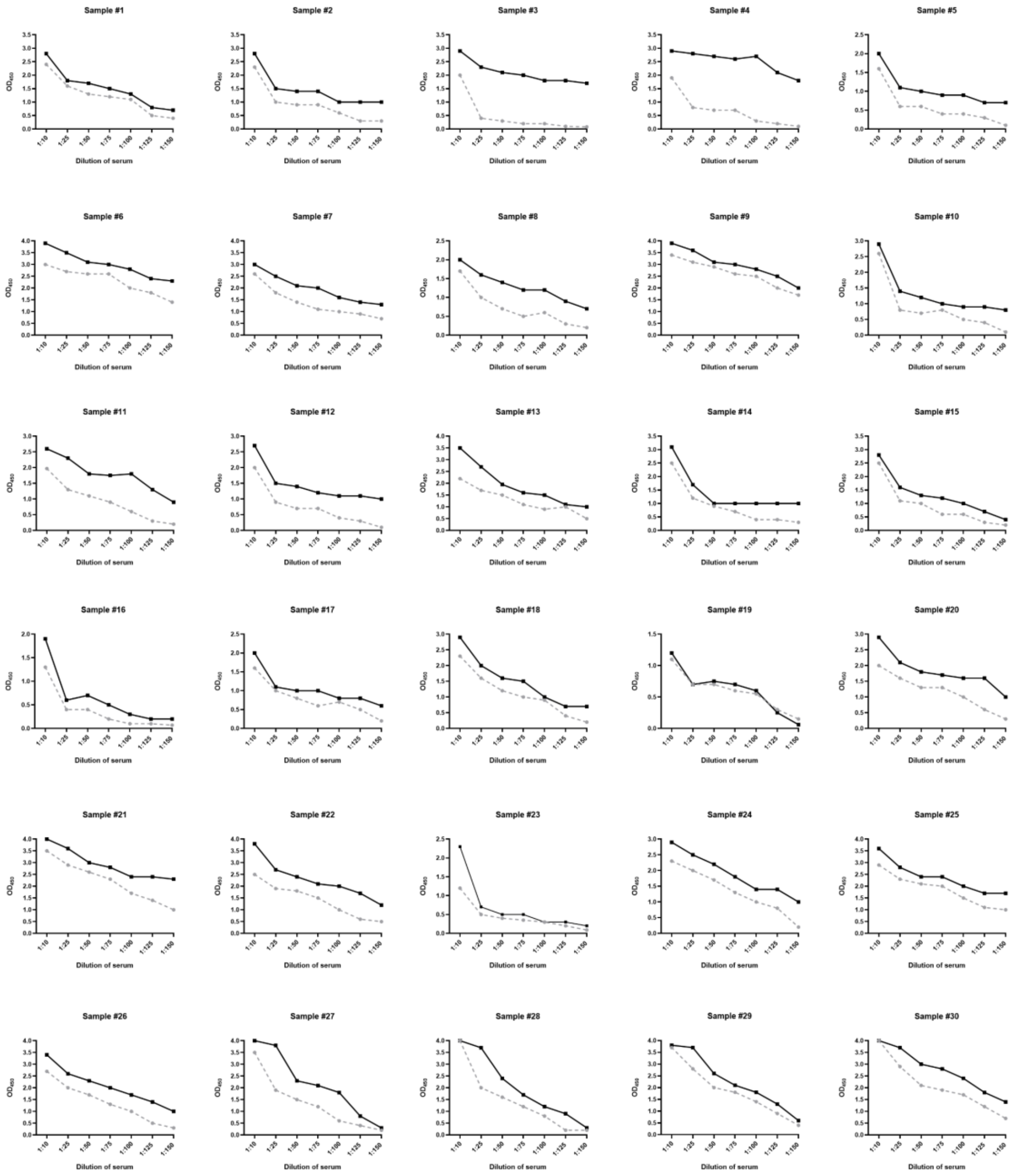

Supplementary Figure 9. Plots depict trends in ELISA data that are maintained over the dilution series tested
People with Cystic Fibrosis as shown in Fig. 3.
